# UPLC-MS/MS Method for the Determination of 14 Compounds in Rat Plasma and Its Application in a Pharmacokinetic Study of Orally Administered Xiaoyao Powder

**DOI:** 10.3390/molecules23102514

**Published:** 2018-09-30

**Authors:** Mingyue Xu, Zhanling Xu, Qingxuan Xu, Hongyue Zhang, Mingyang Liu, Fang Geng, Ning Zhang

**Affiliations:** 1 Key Laboratory of Photochemistry Biomaterials and Energy Storage Materials of Heilongjiang Province, College of Chemistry & Chemical Engineering, Harbin Normal University, No.1 South Road, Limin Development Zone, Harbin 150025, China; xumingyue1116@163.com (M.X.); zhanghongyue80@163.com (H.Z.); 15765566250@163.com (M.L.); 2 College of Jiamusi, Heilongjiang University of Chinese Medicine, Jiamusi, Heilongjiang 154007, China; 15045559036@163.com; 3 Crop Academy of Heilongjiang University, Harbin 150080, China; xuqx@hlju.edu.cn

**Keywords:** Xiaoyao Powder, pharmacokinetics, UPLC-MS/MS, rat plasma, oral administration

## Abstract

Xiaoyao Powder (XYP), a common Chinese medicine, comprises eight traditional Chinese herbs and has been widely used clinically to treat liver damage and mental disorders. An ultra-performance liquid chromatography–tandem mass spectrometry method was developed to investigate the pharmacokinetics of 14 compounds (albiflorin, paeoniflorin, ferulic acid, senkyunolide I, quercetin, isoliquiritigenin, atractylenolide III, ligustilide, atractylenolide II, liquiritin, liquiritigenin, saikosaponin c, glycyrrhizic acid, and saikosaponin a) in XYP. Naringenin was used as the internal standard. The compounds were separated using an ACQUITY UPLC^TM^ BEH C18 column (1.7 μm, 50 × 2.1 mm) with a mobile phase consisting of acetonitrile and 0.1% formic acid in water at a flow rate of 0.3 mL/min. Detection was performed on a triple-quadrupole tandem mass spectrometer using multiple reaction monitoring and an electrospray ionization source in both positive and negative ionization modes. All calibration curves exhibited good linearity (r^2^ > 0.9974) over the measured ranges. The intra- and inter-day precisions were within 12%, and the accuracy ranged from 89.93% to 106.64%. Extraction recovery and matrix effect results were satisfactory. The method was successfully applied in a pharmacokinetic study of the 14 compounds in rat plasma after the oral administration of XYP.

## 1. Introduction

Xiaoyao Powder (XYP), a traditional Chinese medicine formula, was first described in the Taiping Huimin Heji Jufang during the Song Dynasty of China (960–1127 AD). This formula has been widely used to treat liver damage and as an antidepressant for more than 2000 years [1,2,3,4]. According to the Chinese Pharmacopoeia, XYP consists of Radix Bupleuri (*Bupleurum chinense* DC), Radix Angelicae Sinensis (*Angelica sinensis* (Oliv.) Diels), Radix Paeoniae Alba (*Paeonia lactiflora* Pall.), Rhizoma Atractylodis Macrocephalae (*Atractylodes macrocephala* Koidz.), Poria (*Poria cocos* (Schw.) Wolf), Radix Glycyrrhizae (*Glycyrrhiza uralensis* Fisch.), Herba Menthae (*Mentha haplocalyx* Briq.) and Rhizoma Zingiberis Recens (*Zingiber officinale* Rosc.) in a ratio of 5:5:5:5:5:4:1:1 [5].

In our previous study, the components of XYP were identified by ultra-performance liquid chromatography coupled with electrospray ionization tandem mass spectrometry (UPLC-ESI-MS) and gas chromatography-mass spectrometry (GC-MS), yielding a comprehensive list of compounds in XYP [6,7]. XYP and these principle constituents showed sufficient hepatoprotective effects on hepatic fibrosis induced in rats by CCl_4_ [8]. Pharmacokinetics is helpful to evaluate the rationality and compatibility of Chinese herbal medicine or prescription, in terms of efficacy and toxicity [9]. Up to now, there are no reports on which of these XYP compounds are absorbed, nor which may have a key role in the pharmacokinetics of XYP. 

Firstly, a serum pharmacochemistry study was performed, where we tried to determine the components’ plasma absorption after oral administration of XYP to rats. In the pilot study, 14 compounds were identified by UPLC-MS/MS as chemical entities that were absorbed from the gastrointestinal tract after oral administration. Some of these compounds have been reported to be related with hepatoprotective or antidepressant effects. Radix Bupleuri is a principal herb in this formula for liver disorder treatment, and quercetin, saikosaponin a and saikosaponin c from this herb were proved to be the effective ingredients [10,11,12]. Bioactivity studies showed that ligustilide is the major compound in Radix Angelicae Sinensis connected with central nervous system protection [13,14]. As another main compound of Radix Angelicae Sinensis, ferulic acid showed protective activities against CCl_4_-induced liver injury in mice [15]. Liquiritin, liquiritigenin, isoliquiritigenin, and glycyrrhizic acid are the major hepatoprotective components of Radix Glycyrrhizae [16,17,18,19,20]. Paeoniflorin and albiflorin are the most abundant compounds in Radix Paeoniae Alba [21,22,23]. Senkyunolide I can protect nervus centralis against focal cerebral ischemia-reperfusion injury [24]. Rhizoma Atractylodis Macrocephalae has been used in traditional Chinese medicine to treat melancholia. Its main active components are atractylenolide II and atractylenolide III [25,26]. Based on the pilot study described above and our literature review, we determined to study the pharmacokinetic characteristics of these 14 compounds (albiflorin, paeoniflorin, ferulic acid, senkyunolide I, quercetin, isoliquiritigenin, atractylenolide III, ligustilide, atractylenolide II, liquiritin, liquiritigenin, saikosaponin c, glycyrrhizic acid, and saikosaponin a) that are most likely to represent the pharmacokinetic profile of XYP.

Pharmacokinetics studies of some of these 14 compounds in other Chinese formula or single herb have been reported. However, the possibility of interaction between components in an herbal formula and the pharmacokinetic variation of one single compound in different formulas that a multiple-components pharmacokinetic study would be more likely to illustrate the pharmacokinetic profile of XYP. An ultra-performance liquid chromatography–tandem mass spectrometry (UPLC-MS/MS) method is the best and high efficient approach for simultaneous determination of multiple compounds in a complex matrix [27]. Thus, the pharmacokinetic profiles for 14 compounds of XYP were described by UPLC-MS/MS. A multiple reaction monitoring (MRM) pattern was selected as the quantification model to decrease the matrix effect. The pharmacokinetic study of rats after oral administration of XYP fully verified the effectiveness of the method. It is expected that the present work will provide helpful information on the mechanism (s) of action of XYP and understanding of the pharmacokinetics of multiple active components to further the effective clinical application and safety evaluation of Chinese medicines.

## 2. Results and Discussion

### 2.1. Method Validation

#### 2.1.1. Specificity

**Figure 1:**
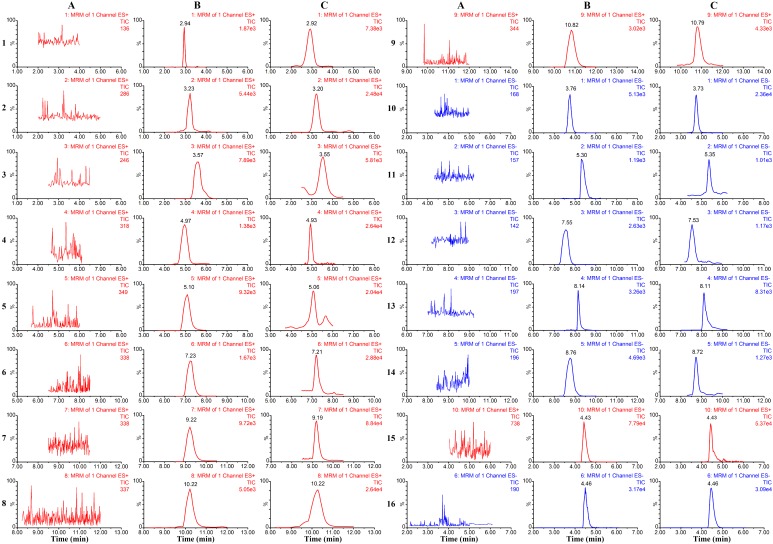
Representative MRM chromatograms of compounds 1–14 and naringenin (IS) in positive ion mode (red) and negative ion mode (blue). (**A**): blank plasma; (**B**): blank plasma spiked with the fifteen analytes and IS; (**C**): plasma sample1h after oral administration of XYP (4 g/kg) (mean ± SD, *n* = 6). (1) albiflorin, (2) paeoniflorin, (3) quercetin, (4) ferulic acid, (5) senkyunolide I, (6) isoliquiritigenin, (7) atractylenolide III, (8) ligustilide, (9) atractylenolide II, (10) liquiritin, (11) liquiritigenin, (12) saikosaponin c, (13) glycyrrhizic acid, (14) saikosaponin a, (15) naringenin (IS) in positive ion mode and (16) naringenin (IS) in negative ion mode.

The representative chromatograms of blank plasma, blank plasma spiked with reference standards and IS, and plasma obtained after the oral administration of XYP are shown in Figure 1. Under the established optimal chromatographic conditions, no significant interfering peaks were observed at the analyte elution times, and no interference occurred between the IS and the 14 analytes.

#### 2.1.2. Linearity and Calibration Curve

The linearity regression equation, correlation coefficients, and linear ranges of the 14 analytes are shown in Table 1. Good linearity was obtained for all analytes. The LLOQ values were appropriate for the quantitative detection of the analytes in pharmacokinetic studies.

#### 2.1.3. Precision and Accuracy

The precision and accuracy of the UPLC-MS/MS method (Table 2) were within acceptable limits. The intra-day accuracy ranged from 90.39–106.52% with a precision of 1.21–11.71%. The inter-day accuracy was 89.93–106.64% with a precision of 2.33–11.55%.

#### 2.1.4. Extraction Recovery and Matrix Effect

Matrix effects and recovery in Table 3 show that no endogenous substance in plasma has a significant effect on the ionization of the analyte. The matrix effects were within an acceptable range 85.17% to 102.69%, and the mean extraction recoveries of the 14 analytes and IS were greater than 82.33%.

#### 2.1.5. Stability

The results of stability experiments (Table 4) showed that no significant degradation occurred. The concentrations of the 14 analytes measured in the stability study were within 7.45% of the initial values. The data indicated that all analytes in rat plasma were stable after storage at −20 °C for 30 days, after three freeze/thaw cycles, and after storage in the auto-sampler (4 °C) for 36 h.

#### 2.1.6. Pharmacokinetic Study

**Figure 2:**
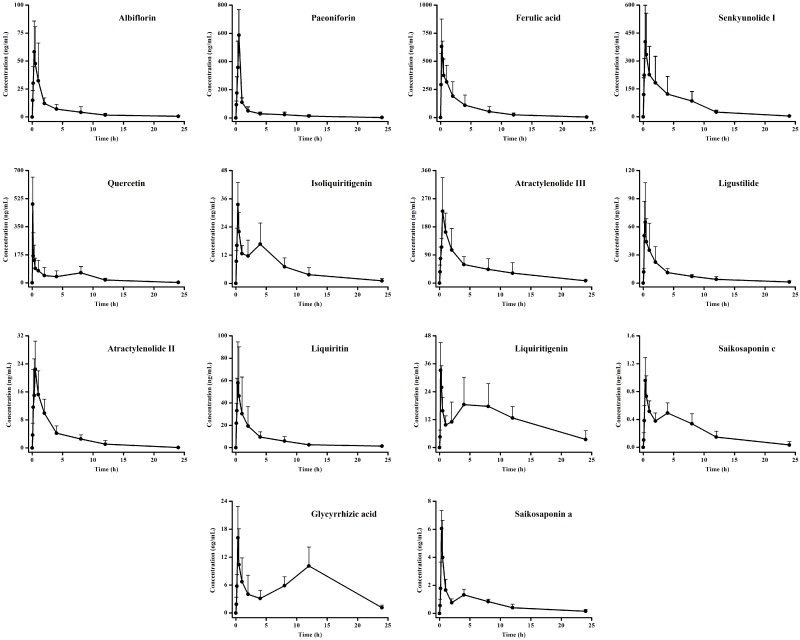
Plasma concentration-time profiles of 14 analytes in rats after oral administration of XYP (4 g/kg) (mean ± SD, *n* = 6).

The validated UPLC-MS/MS method was successfully applied to the pharmacokinetic study of 14 compounds in rat plasma after the oral administration of XYP at a dose of 4 g/kg (8-fold the clinical dosage). The mean plasma concentration–time profiles are shown in Figure 2. Pharmacokinetic parameters were calculated and are presented in Table 5.

Paeoniflorin and albiflorin are representative compounds of Radix Paeoniae Alba. The absorption and elimination of paeoniflorin in rat plasma were faster, with more absorption and higher bioavailability than that of albiflorin. Ferulic acid was detected in plasma at 5 min and the plasma concentration reached its peak (C_max_) at 0.2 h (T_max_) for rats (Figure 2), which was very quick and similar to the results found in the literature [28]. The t_max_ of atractylenolide II and atractylenolide III were similar due to their similar structures. However, atractylenolide II and atractylenolide III showed significant differences in their absorption parameters. The concentrations of the two compounds in XYP extract were similar, but higher levels in the AUC_0–t_ and C_max_ of atractylenolide III were observed, compared with atractylenolide II after intragastric administration of XYP extract. The significant differences existed in the absorption of the PK parameters between atractylenolide II and III, which is correspondence with a pharmacokinetic study of atractylenolide II and III in rat plasma after intragastric administration of Baizhufuling extract. This phenomenon may be caused by the higher polarity and slower elimination rate (t_1/2_) of atractylenolide III [29,30]. Quercetin is a widely used dietary flavonoid, which can be directly absorbed into epithelial cells in the intestine to enter the circulatory system finally. Therefore, it can be more easily and faster absorbed in plasma with t_max_ of 0.1 h. The pharmacokinetic profile of liquiritin was similar to a previous report [31,32]. Liquiritin and senkyunolide I were rapidly absorbed and eliminated after oral administration. Compared with previous study [33,34], we found that they showed similar pharmacokinetic profile. The study indicated that ligustilide reached a maximum concentration within approximately 0.3 h and the pharmacokinetic profile of ligustilide was accordance with a previous research [35].

As flavonoid aglycones, liquiritigenin, isoliquiritigenin and quercetin reached the second maximal plasma concentrations at about 4 h, 4 h and 8 h. Saikosaponin a, saikosaponin c and glycyrrhizic acid are triterpene glycosides with double peaks in the plasma concentration-time curves as well. This might be attributed to some factors that include enterohepatic recirculation, variable gastric emptying, multiple sites absorption, formulation, etc. The first pass effect of liver and intestine probably contributed to the high concentration double peaks as well [36,37]. Several reasons could explain the double peak phenomenon of flavonoid aglycones. Firstly, the phenomenon maybe caused with enterohepatic recirculation. The enterohepatic circulation of flavonoids has been reported in serval studies. Secondly, the flavonoid glycosides were probably hydrolyzed to flavonoid aglycones by glucose hydrolase in vivo. Thus, second absorption peaks of flavonoid aglycones existed. The double peak plasma concentration-time curves of liquiritigenin, isoliquiritigenin and liquiritin are identical to the literature ones [33,38].

## 3. Materials and Methods

### 3.1. Chemicals and Reagents

**Figure 3:**
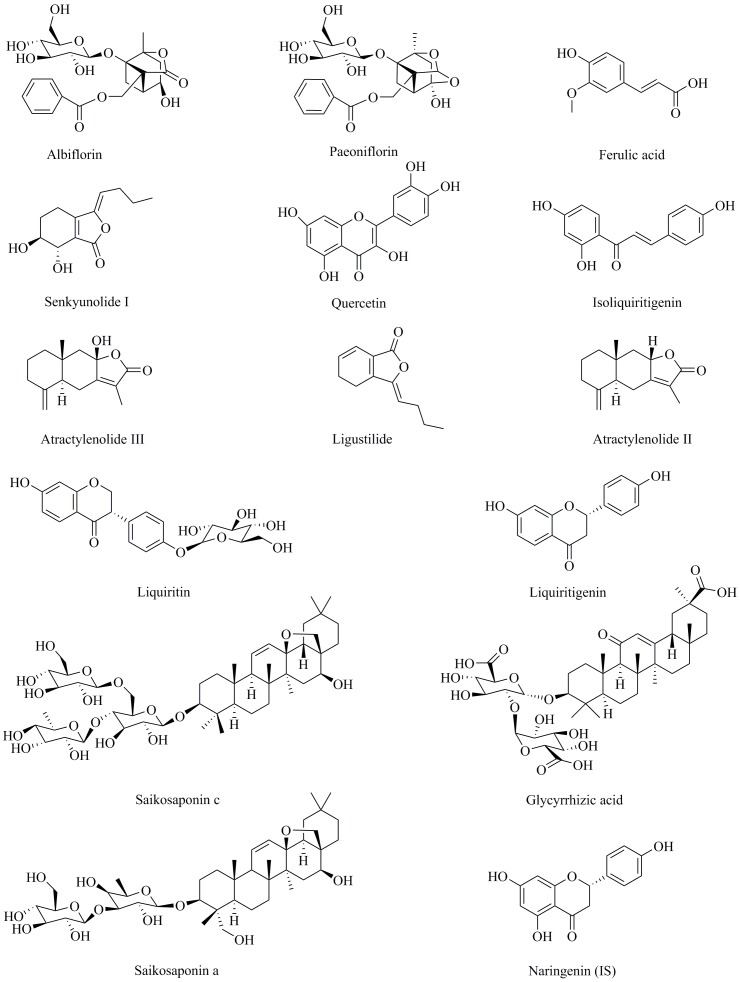
Chemical structures of the fourteen analytes and the IS (naringenin).

Standard compounds of albiflorin, paeoniflorin, ferulic acid, senkyunolide I, quercetin, isoliquiritigenin, atractylenolide III, ligustilide, atractylenolide II, liquiritin, liquiritigenin, saikosaponin c, glycyrrhizic acid, saikosaponin a and naringenin as an internal standard (IS) (≥98% purity) were purchased from Chengdu Herbpurify Co., Ltd. (Chengdu, China). The structures of these compounds are shown in Figure 3. 

Acetonitrile (HPLC grade) was purchased from Fisher (Fair Lawn, NJ, USA). Ultrapure water with a sensitivity of 18.2 W was prepared from a Milli-Q water purification system (Millipore, Billerica, MA, USA). All other chemicals were analytical grade.

### 3.2. Instrumentation and Ultraperformance Liquid Chromatography-Tandem Mass Spectrometry (UPLC-MS/MS) Analytical Conditions

Quantitative analysis was conducted on a Waters^®^ Micromass^®^ Quattro Premier™ XE triple-quadrupole tandem mass spectrometer (Waters Corp., Milford, MA, USA). Chromatographic separation was performed on an ACQUITY UPLCTM BEH C18 column (1.7 µm, 50 × 2.1 mm; Waters Corp.) equipped with an ACQUITY UPLC C18 guard column. The column temperature was maintained at 35 °C. The mobile phase consisted of 0.1% formic acid in water (A) and acetonitrile (B). The total flow rate was 0.3 mL/min. Chromatographic separation was achieved using a 14.5-min gradient elution. The gradient conditions of the mobile phase were as follows: 0–4 min, 4–22% (B); 4–5 min, 22–27% (B); 5–10 min, 27–50% (B); and 10–14.5 min, 50–96% (B). The samples were kept at 4 °C in the auto-sampler, and the injection volume was 5 µL.

The mass spectrometer was operated using an electrospray ionization source in positive and negative ion modes. Quantification was performed in positive or negative MRM mode. The MS parameters for nine compounds (albiflorin, paeoniflorin, ferulic acid, senkyunolide I, quercetin, isoliquiritigenin, atractylenolide III, ligustilide, and atractylenolide II) were optimized in the positive mode, whereas the MS parameters for the other five compounds (liquiritin, liquiritigenin, saikosaponin c, glycyrrhizic acid, and saikosaponin a) were optimized under negative ion mode. The parameters of the mass spectrometer were set as follows: capillary voltage, 3.50 kV (+) and 3.0 kV (−); source temperature, 120 °C; and desolvation temperature 300 °C. Argon was used as the collision gas at a pressure of approximately 2.89 × 10^−3^ mbar. Nitrogen was used as the desolvation and cone gas with flow rates of 600 and 50 L/h, respectively. In Table 6, the cone voltage and collision energy of each compound were optimized. The dwell time was set as 0.2 s. All data collected were processed using Mass Lynx™ NT 4.1 software with the Quan Lynx™ program (Waters, Milford, MA, USA).

### 3.3. Preparation of Standards and Quality Control (QC) Samples

Stock solutions (100 µg/mL) of albiflorin, paeoniflorin, ferulic acid, senkyunolide I, quercetin, isoliquiritigenin, atractylenolide III, ligustilide, atractylenolide II, liquiritin, liquiritigenin, saikosaponin c, glycyrrhizic acid, saikosaponin a and naringenin (IS) were separately prepared in methanol. The stock solutions of analytes were further mixed and diluted with methanol to yield standard working solutions. The concentrations of the working solutions ranged from 0.46–480 ng/mL for albiflorin, 1.02–1080 ng/mL for paeoniflorin, 2.08–1100 ng/mL for ferulic acid, 2.70–1150 ng/mL for senkyunolide I, 2.15–1085 ng/mL for quercetin, 0.54–485 ng/mL for isoliquiritigenin, 1.05–1105 ng/mL for atractylenolide III, 1.44–1050 ng/mL for ligustilide, 0.51–480 ng/mL for atractylenolide II, 0.53–495 ng/mL for liquiritin, 1.13–1050 ng/mL for liquiritigenin, 0.48–510 ng/mL for saikosaponin c, 0.96–1120 ng/mL for glycyrrhizic acid, and 0.49–510 ng/mL for saikosaponin a. The working solution of IS was prepared by diluting the stock solution to a final concentration of 10 μg/mL using methanol. Three levels of QC samples (low, intermediate and high) were prepared in the same manner. All samples were stored at −20 °C.

### 3.4. Preparation and Quality Assessment of XYP Extract

The crude plant materials purchased at Tongren Tang. Professor Zhang Ning of Heilongjiang University of Traditional Chinese Medicine identify correct plants by the Chinese Pharmacopoeia 2015. The Identification results were preserved in Heilongjiang University of Traditional Chinese Medicine. The crude powders of Radix Bupleuri (100 g), Radix Angelicae Sinensis (100 g), Radix Paeoniae Alba (100 g), Rhizoma Atractylodis Macrocephalae (100 g), Poria (100 g), Radix Glycyrrhizae (80 g), Herba Menthae (20 g), and Rhizoma Zingiberis Recens (20 g) were mixed together and refluxed with 60% ethanol for 2 h three times. The filtered decoctions are merged, evaporated under reduce pressure at 50 °C and lyophilized with a yield of 26.1%. To calculate the administration dose, the 14 target compounds in XYP extract were determined by UPLC-MS/MS.

The dried extract was dissolved in 70% methanol with a solid–liquid ratio of 30:1 mg/mL (*w*/*v*), after centrifugation, supernatant was injected into the detector. Then, the content is calculated using the obtained standard curve. The quantitative result showed the contents of albiflorin, paeoniflorin, ferulic acid, senkyunolide I, quercetin, isoliquiritigenin, atractylenolide III, ligustilide, atractylenolide II, liquiritin, liquiritigenin, saikosaponin c, glycyrrhizic acid, saikosaponin a in XYP extract to be 0.62, 1.7, 5.46, 1.45, 0.70, 0.13, 1.42, 0.14, 1.01, 1.42, 1.23, 0.10, 1.12, 0.09 mg/g, respectively.

### 3.5. Plasma Sample Preparation

Methanol (1.5 mL), 10 μg/mL IS and 300 μL plasma was mixed, then vortexed for 1 min. After centrifuging at 14,000× *g* for 10 min, the supernatant was put into another centrifuge tube. The residue was vortexed with another 1.5 mL methanol and centrifuged again, and the two supernatants were combined and evaporated to dryness under a flow of nitrogen gas at 40 °C. The residue was then dissolved in 100 μL methanol and vortexed for 1 min. Finally, the vortexing and centrifugation process was again repeated, and 5 μL of the supernatant was injected into the UPLC-MS/MS system for analysis.

### 3.6. Method Validation

#### 3.6.1. Specificity

Specificity was assessed by a comparing the chromatograms of blank plasma, blank plasma with the 14 compounds and IS and a plasma sample obtained after the oral administration of XYP.

#### 3.6.2. Linearity and Lower Limit of Quantitation (LLOQ)

The calibration curves were constructed by the plot of the peak area ratios of the analytes versus the IS against the concentrations of the standards and weight coefficient was 1/x^2^. The LLOQ was defined as the lowest concentration on the standard curve that could be quantitated with an accuracy of 80–120% and a precision (relative standard deviation, RSD, %) value not exceeding 20%.

#### 3.6.3. Precision and Accuracy

Intra- and inter-day accuracy and precision were evaluated from replicate analyses (*n* = 6) of XYP samples containing different concentrations of analytes (low, intermediate, and high). The results confirm that the proposed method provides satisfactory accuracy and precision for the quantitation of all 14 analytes.

#### 3.6.4. Extraction Recovery and Matrix Effect

The extraction recoveries of the analytes at the three QC levels were determined by comparing the peak areas obtained from 14 extracted QC samples with those obtained from pure reference standards spiked into post-extracted blank rat plasma at the same concentrations. The matrix effects were evaluated by comparing the peak areas obtained from samples for which the extracted matrix was spiked with a standard solution to those obtained from a pure reference standard solution at the same concentrations.

#### 3.5.5. Stability

The stabilities (expressed as relative error, RE, %) of the 14 analytes in plasma were assessed by analyzing the samples with three concentrations (high, intermediate, and low) during the sample storage and processing procedures. The short-term stability was assessed by analyzing the QC samples kept in the auto-sampler (4 °C) for 36 h. To assess long-term stability, the QC samples were stored at −20 °C for 30 days. Freeze-thaw stability was evaluated by subjecting the QC plasma samples to three complete freeze/thaw cycles from −20 °C to room temperature.

### 3.7. Pharmacokinetic Study

Twenty healthy female Sprague–Dawley rats (300 ± 20 g) were purchased from the Animal Safety Evaluation Center of Heilongjiang University of Chinese Medicine (Heilongjiang, China). All protocols for animal experiments were approved in accordance with the Regulations of Experimental Animal Administration issued by the State Commission of Science and Technology of the People’s Republic of China. The rats were kept under a light/dark cycle of 12 h, within the temperature range of 24 °C ± 2 °C to room temperature, and at a humidity of 50 ± 10%. All rats were fasted for 12 h before experimentation but were allowed to drink freely. XYP extract was dissolved in water, and the rats were administrated orally with 4 g/kg XYP. Blood samples were collected from the suborbital venous lexus at 0, 0.083, 0.17, 0.33, 0.50, 0.75, 1.0, 2.0, 4.0, 8.0, 12 and 24 h into 1.5-mL heparinized polythene tubes before dosing. The blood samples were immediately centrifuged at 4500 rpm for 10 min, and the plasma was stored at −80 °C until analysis. The pharmacokinetic parameters (Cmax, t_max_, t_1/2_, Kel, AUC_0–t_, AUC_0–∞_, MRT_0–t_, MRT_0–∞_) were calculated with the software Winnolin 3.2.

## 4. Conclusions

A sensitive and selective UPLC-MS/MS method was developed and applied in a multi-component pharmacokinetic study of XYP administered orally to rats at a dose of 4 g/kg. The developed method can be used to monitor albiflorin, paeoniflorin, ferulic acid, senkyunolide I, quercetin, isoliquiritigenin, atractylenolide III, ligustilide, atractylenolide II, liquiritin, liquiritigenin, saikosaponin c, glycyrrhizic acid and saikosaponin a in rat plasma. The compounds of different structural types (saponins, flavonoids, terpenoids, phenylpropionic acids, coumarin and phthalide) exhibited characteristic pharmacokinetic behaviors. The UPLC-MS/MS method represents the first documented assay for the pharmacokinetic determination of the multiple active ingredients in XYP. This method is expected to be valuable to identify bioactive constituents, elucidate mechanisms of action, and guide the rational clinical use of herbal medicines.

## Figures and Tables

**Table 1:** Regression equations, linearity and LLOQ.

Analytes	Regression Equation	Correlation Coefficient (r^2^)	Linear Range (ng/mL)	LLOQ (ng/mL)
Albiflorin	y = 8.9467x + 43.276	0.9996	0.46–480	0.46
Paeoniflorin	y = 2.8358x + 51.224	0.9999	1.02–1080	1.02
Ferulic acid	y = 1.6548x + 0.5608	0.9989	2.08–1100	2.08
Senkyunolide I	y = 3.4788x − 1.5320	0.9997	2.70–1150	2.70
Quercetin	y = 1.9983x + 8.4101	0.9974	2.15–1085	2.15
Isoliquiritigenin	y = 44.035x + 25.188	0.9993	0.54–485	0.54
Atractylenolide III	y = 30.767x − 9.5428	0.9999	1.05–1105	1.05
Ligustilide	y = 2.3836x + 33.177	0.9989	1.44–1050	1.44
Atractylenolide II	y = 20.035x + 48.474	0.9990	0.51–480	0.51
Liquiritin	y = 17.470x + 52.527	0.9995	0.53–495	0.53
Liquiritigenin	y = 0.3824x + 5.4505	0.9993	1.13–1050	1.13
Saikosaponin c	y = 38.923x + 39.843	0.9988	0.48–510	0.48
Glycyrrhizic acid	y = 47.474x + 24.225	0.9994	0.96–1120	0.96
Saikosaponin a	y = 39.303x + 37.598	0.9990	0.49–510	0.49

LLOQ: lower limit of quantification.

**Table 2:** Intra-day and inter-day precision and accuracy (*n* = 6).

Analytes	Normal Concentration (ng/mL)	Intra-Day			Inter-Day		
		Concentration Measured (ng/mL)	Precision (RSD%)	Accuracy (%)	Concentration Measured (ng/mL)	Precision (RSD%)	Accuracy (%)
Albiflorin	3.8	3.86 ± 0.17	4.38	101.67	3.66 ± 0.22	6.06	96.18
	38.0	36.66 ± 2.77	7.55	96.48	39.20 ± 2.23	5.70	103.16
	380.0	385.32 ± 23.40	6.07	101.40	388.22 ± 15.25	3.93	102.16
Paeoniflorin	5.1	4.66 ± 0.39	8.33	91.34	4.94 ± 0.25	4.98	96.76
	510.0	495.77 ± 6.01	1.21	97.21	491.59 ± 17.60	3.58	96.39
	1020.0	1032.32 ± 36.04	3.49	101.21	1065.80 ± 70.41	6.61	104.49
Ferulic acid	5.1	5.10 ± 0.15	2.89	100.96	5.11 ± 0.13	2.60	101.22
	505.0	502.84 ± 29.69	5.90	99.57	486.81 ± 15.09	3.10	96.40
	1010.0	1037.86 ± 84.16	8.11	102.76	1039.58 ± 102.41	9.85	102.93
Senkyunolide I	4.2	4.13 ± 0.16	3.94	98.21	4.08 ± 0.19	4.65	97.02
	42.0	39.15 ± 2.50	6.39	93.21	42.49 ± 2.41	5.67	101.17
	840.0	778.87 ± 46.41	5.96	92.72	846.56 ± 35.76	4.22	100.78
Quercetin	5.0	4.95 ± 0.15	2.98	98.15	4.94 ± 0.13	2.62	98.05
	504.0	474.88 ± 41.79	8.80	94.22	494.74 ± 11.51	2.33	98.16
	1008.0	1005.27 ± 43.45	4.32	99.73	980.91 ± 26.41	2.69	97.31
Isoliquiritigenin	3.8	3.71 ± 0.18	4.98	98.89	3.46 ± 0.16	4.55	92.31
	37.5	39.22 ± 1.65	4.21	104.59	39.25 ± 2.02	5.14	104.66
	375.0	370.56 ± 43.39	11.71	98.82	390.86 ± 16.99	4.35	104.23
Atractylenolide III	4.5	4.19 ± 0.25	5.93	93.19	4.23 ± 0.41	9.73	93.89
	90.0	93.92 ± 9.80	10.43	104.35	95.97 ± 3.44	3.58	106.64
	900.0	958.69 ± 59.32	6.19	106.52	931.56 ± 48.21	5.18	103.51
Ligustilide	5.2	4.94 ± 0.15	3.06	95.03	4.86 ± 0.56	11.55	93.40
	104.0	94.37 ± 8.39	8.89	90.74	96.37 ± 4.44	4.60	92.66
	1040.0	940.05 ± 67.12	7.14	90.39	973.42 ± 57.66	5.92	93.60
Atractylenolide II	2.4	2.33 ± 0.14	5.91	97.01	2.24 ± 0.08	3.42	93.19
	48.0	47.12 ± 1.55	3.29	98.17	49.78 ± 1.92	3.87	103.70
	480.0	469.78 ± 33.54	7.14	97.87	458.48 ± 24.18	5.27	95.52
Liquiritin	2.1	1.94 ± 0.09	4.48	94.72	2.01 ± 0.11	5.39	98.21
	41.0	39.30 ± 1.56	3.96	95.85	40.67 ± 3.16	7.76	99.19
	410.0	383.02 ± 28.28	7.38	93.42	391.23 ± 20.06	5.13	95.42
Liquiritigenin	4.5	4.20 ± 0.28	6.79	93.26	4.05 ± 0.28	6.96	89.93
	90.0	93.48 ± 5.16	5.52	103.87	93.71 ± 2.87	3.06	104.12
	900.0	952.35 ± 52.75	5.54	105.82	959.66 ± 71.44	7.44	106.63
Saikosaponin c	1.2	1.16 ± 0.06	5.58	96.25	1.21 ± 0.06	5.02	101.11
	24.0	22.33 ± 2.09	9.37	93.02	23.34 ± 2.08	8.89	97.26
	480.0	504.04 ± 11.29	2.24	105.01	493.36 ± 13.73	2.78	102.78
Glycyrrhizic acid	4.6	4.24 ± 0.34	8.03	92.25	4.32 ± 0.30	6.90	93.91
	460.0	430.82 ± 24.47	5.68	93.66	434.50 ± 27.53	6.34	94.46
	920.0	910.12 ± 33.66	3.70	98.93	935.77 ± 22.66	2.42	101.71
Saikosaponin a	1.3	1.20 ± 0.06	5.20	94.23	1.17 ± 0.07	5.96	91.99
	25.5	26.78 ± 1.18	4.42	105.01	23.51 ± 2.00	8.51	92.18
	510.0	491.71 ± 14.57	2.96	96.41	496.81 ± 15.44	3.11	97.41

RSD%: expressed as relative standard deviation.

**Table 3:** Extraction recovery and matrix effect at low, intermediate and high concentration levels (mean ± SD, *n* = 6).

Analyte	Normal Concentration (ng/mL)	Extraction Recovery (%)	Matrix Effect (%)
Albiflorin	3.8	101.11 ± 2.90	95.28 ± 4.49
	38	101.49 ± 4.99	93.33 ± 2.71
	380	95.48 ± 6.14	93.81 ± 4.46
Paeoniflorin	5.1	94.74 ± 4.63	92.58 ± 3.26
	510	101.03 ± 5.86	95.36 ± 4.47
	1020	94.73 ± 5.98	94.54 ± 3.24
Ferulic acid	5.1	94.23 ± 5.33	91.89 ± 4.10
	505	96.97 ± 3.00	86.75 ± 2.97
	1010	99.97 ± 5.06	98.31 ± 5.77
Senkyunolide I	4.2	105.77 ± 6.42	97.43 ± 4.98
	42	103.25 ± 3.04	93.14 ± 3.80
	840	109.75 ± 7.55	97.42 ± 5.82
Quercetin	5	88.84 ± 2.40	88.56 ± 2.23
	504	88.58 ± 3.61	95.25 ± 6.04
	1008	88.58 ± 3.61	86.95 ± 3.81
Isoliquiritigenin	3.8	95.24 ± 6.57	94.24 ± 6.16
	37.5	103.67 ± 5.20	97.77 ± 2.90
	375	97.68 ± 7.33	94.35 ± 3.53
Atractylenolide III	4.5	94.92 ± 7.83	91.59 ± 6.30
	90	87.21 ± 4.50	88.21 ± 3.10
	900	95.53 ± 7.38	95.77 ± 7.23
Ligustilide	5.2	87.68 ± 5.09	87.01 ± 3.67
	104	85.53 ± 3.58	85.79 ± 3.37
	1040	85.48 ± 3.99	86.81 ± 3.42
Atractylenolide II	2.4	101.48 ± 7.06	98.42 ± 5.00
	48	99.79 ± 3.81	94.96 ± 7.54
	480	103.41 ± 7.25	98.41 ± 3.62
Liquiritin	2.1	107.64 ± 7.48	98.62 ± 4.87
	41	104.79 ± 2.57	94.62 ± 5.85
	410	111.77 ± 2.80	102.69 ± 7.25
Liquiritigenin	4.5	87.65 ± 4.41	87.32 ± 4.43
	90	99.49 ± 7.97	99.49 ± 7.97
	900	99.49 ± 7.97	94.66 ± 4.64
Saikosaponin c	1.2	100.43 ± 6.77	92.73 ± 3.79
	24	99.35 ± 7.45	93.85 ± 3.95
	480	95.81 ± 8.53	95.81 ± 8.53
Glycyrrhizic acid	4.6	82.33 ± 2.97	85.17 ± 4.42
	460	85.67 ± 2.67	87.47 ± 2.05
	920	85.18 ± 3.17	86.52 ± 4.03
Saikosaponin a	1.3	93.02 ± 6.79	92.69 ± 6.93
	25.5	88.10 ± 4.68	87.31 ± 4.75
	510	90.20 ± 6.28	91.13 ± 5.58

**Table 4:** Stability evaluation results (*n* = 6).

Analytes	Spiked (ng/mL)	Auto-Sampler (4 °C, 36 h)		Long-Term (−20 °C, 30 Days)		Freeze-Thaw (−20 °C-Room Temperature)	
		Measured	RE (%)	Measured	RE (%)	Measured	RE (%)
Albiflorin	3.80	3.91 ± 0.27	2.81	3.76 ± 0.50	−1.18	3.65 ± 0.06	−4.04
	38.00	37.83 ± 2.50	−0.45	38.87 ± 1.83	2.29	37.00 ± 2.32	−2.64
	380.00	389.79 ± 11.12	2.58	386.55 ± 12.40	1.72	385.16 ± 23.24	1.36
Paeoniflorin	5.10	5.02 ± 0.18	−1.63	4.99 ± 0.07	−2.19	5.01 ± 0.21	−1.80
	510.00	497.41 ± 11.78	−2.47	501.59 ± 11.11	−1.65	494.11 ± 8.68	−3.12
	1020.00	1056.41 ± 30.28	3.57	1032.46 ± 35.15	1.22	1030.65 ± 34.59	1.04
Ferulic acid	5.05	5.10 ± 0.06	0.92	5.10 ± 0.11	0.89	5.08 ± 0.11	0.66
	505.00	481.27 ± 13.64	−4.70	491.81 ± 7.89	−2.61	486.01 ± 17.15	−3.76
	1010.00	1000.91 ± 21.30	−0.90	1006.25 ± 27.81	−0.37	1016.19 ± 45.28	0.61
Senkyunolide I	4.20	4.10 ± 0.13	−2.46	4.09 ± 0.13	−2.58	4.09 ± 0.09	−2.58
	42.00	41.50 ± 3.05	−1.20	41.33 ± 3.09	−1.60	41.13 ± 3.11	−2.07
	840.00	834.09 ± 23.38	−0.70	837.06 ± 26.59	−0.35	824.21 ± 18.77	−1.88
Quercetin	5.04	4.95 ± 0.14	−1.85	4.93 ± 0.13	−2.28	4.94 ± 0.14	−1.95
	504.00	494.39 ± 10.55	−1.91	493.07 ± 13.07	−2.17	491.55 ± 18.55	−2.47
	1008.00	990.24 ± 31.42	−1.76	987.58 ± 36.41	−2.03	986.94 ± 24.22	−2.09
Isoliquiritigenin	3.75	3.75 ± 0.16	−0.13	3.71 ± 0.16	−1.02	3.64 ± 0.34	−2.89
	37.50	37.85 ± 1.42	0.94	38.08 ± 1.50	1.55	37.39 ± 1.90	−0.30
	375.00	371.62 ± 13.67	−0.90	377.52 ± 13.03	0.67	364.56 ± 15.01	−2.78
Atractylenolide III	4.50	4.48 ± 0.16	−0.37	4.41 ± 0.14	−2.11	4.44 ± 0.15	−1.26
	90.00	91.66 ± 3.92	1.85	90.31 ± 2.21	0.34	92.25 ± 3.91	2.50
	900.00	920.70 ± 44.64	2.30	919.90 ± 34.37	2.21	925.35 ± 39.89	2.82
Ligustilide	5.20	5.05 ± 0.13	−2.82	5.02 ± 0.32	−3.40	5.09 ± 0.15	−2.08
	104.00	101.10 ± 4.29	−2.79	99.70 ± 3.76	−4.14	102.04 ± 4.00	−1.89
	1040.00	1023.55 ± 34.04	−1.58	1028.42 ± 35.42	−1.11	1023.38 ± 30.28	−1.60
Atractylenolide II	2.40	2.37 ± 0.27	−1.25	2.49 ± 0.31	3.61	2.36 ± 0.10	−1.60
	48.00	46.05 ± 4.17	−4.06	48.45 ± 6.78	0.93	47.29 ± 1.81	−1.48
	480.00	478.79 ± 24.29	−0.25	465.14 ± 15.69	−3.10	478.11 ± 25.41	−0.39
Liquiritin	2.05	1.96 ± 0.06	−4.39	1.96 ± 0.06	−4.23	1.99 ± 0.07	−2.85
	41.00	40.59 ± 1.99	−1.01	40.50 ± 2.82	−1.22	39.80 ± 1.92	−2.93
	410.00	409.01 ± 8.13	−0.24	407.90 ± 9.65	−0.51	409.52 ± 11.42	−0.12
Liquiritigenin	4.50	4.50 ± 0.12	−0.07	4.35 ± 0.26	−3.41	4.41 ± 0.10	−1.93
	90.00	90.20 ± 3.79	0.22	89.87 ± 3.18	−0.14	91.81 ± 3.04	2.01
	900.00	910.32 ± 16.62	1.15	919.66 ± 9.21	2.18	912.35 ± 24.55	1.37
Saikosaponin c	1.20	1.15 ± 0.14	−4.03	1.20 ± 0.09	−0.28	1.19 ± 0.18	−1.11
	24.00	23.48 ± 1.37	−2.15	24.01 ± 2.90	0.04	23.49 ± 2.06	−2.12
	480.00	492.33 ± 8.81	2.57	491.69 ± 4.90	2.44	491.04 ± 9.82	2.30
Glycyrrhizic acid	4.60	4.62 ± 0.25	0.43	4.57 ± 0.19	−0.65	4.54 ± 0.25	−1.23
	460.00	459.19 ± 22.29	−0.18	462.84 ± 28.06	0.62	447.49 ± 23.22	−2.72
	920.00	920.55 ± 40.71	0.06	930.77 ± 22.18	1.17	910.12 ± 33.66	−1.07
Saikosaponin a	1.27	1.32 ± 0.04	3.54	1.27 ± 0.09	−0.13	1.26 ± 0.10	−1.05
	25.50	27.40 ± 3.34	7.45	25.17 ± 3.20	−1.29	26.19 ± 3.61	2.72
	510.00	501.35 ± 16.88	−1.70	508.81 ± 8.83	−0.23	486.71 ± 15.18	−4.57

RE%: expressed as relative error.

**Table 5:** The main pharmacokinetic parameters after oral administration of XYP with 4 g/kg. Data are presented as mean ± SD (*n* = 6).

Analytes	Parameters							
	t_max_ (h)	C_max_ (ng/mL)	Kel	t_1/2_ (h)	AUC_0–t_ (ng h/mL)	AUC_0–∞_ (ng h/mL)	MRT_0–t_ (h)	MRT_0–∞_ (h)
Albiflorin	0.39 ± 0.09	58.28 ± 27.50	0.13 ± 0.06	7.11 ± 4.57	127.67 ± 64.94	135.02 ± 65.64	4.18 ± 1.58	6.34 ± 4.50
Paeoniflorin	0.42 ± 0.09	586.92 ± 180.66	0.16 ± 0.06	4.82 ± 1.71	696.90 ± 210.89	717.61 ± 227.27	4.17 ± 1.38	4.91 ± 1.68
Ferulic acid	0.21 ± 0.10	632.47 ± 244.04	0.15 ± 0.05	5.30 ± 1.85	1584.14 ± 850.88	1611.48 ± 852.75	3.97 ± 0.80	4.66 ± 0.52
Senkyunolide I	0.36 ± 0.13	403.26 ± 201.00	0.18 ± 0.07	4.75 ± 2.78	1582.38 ± 985.86	1616.46 ± 967.01	5.31 ± 0.72	6.37 ± 2.39
Quercetin	0.10 ± 0.03	491.43 ± 167.80	0.19 ± 0.07	4.09 ± 1.24	766.28 ± 410.22	781.69 ± 415.12	6.40 ± 0.85	7.03 ± 1.02
Isoliquiritigenin	0.39 ± 0.09	33.58 ± 9.37	0.17 ± 0.12	5.82 ± 3.25	156.61 ± 76.73	185.17 ± 89.88	5.98 ± 1.50	7.81 ± 3.39
Atractylenolide III	0.67 ± 0.26	229.66 ± 107.94	0.11 ± 0.05	6.95 ± 2.65	1044.70 ± 496.68	1125.88 ± 487.04	6.23 ± 1.55	8.60 ± 3.13
Ligustilide	0.31 ± 0.13	64.97 ± 42.21	0.17 ± 0.10	5.44 ± 3.09	194.11 ± 57.72	207.32 ± 58.47	5.63 ± 2.32	7.47 ± 4.23
Atractylenolide II	0.64 ± 0.29	22.48 ± 8.04	0.21 ± 0.07	3.54 ± 1.21	69.53 ± 25.72	70.15 ± 25.63	4.15 ± 0.82	4.43 ± 0.98
Liquiritin	0.39 ± 0.09	57.93 ± 36.64	0.09 ± 0.04	9.32 ± 5.49	163.02 ± 91.45	180.94 ± 90.94	5.70 ± 1.25	10.17 ± 6.79
Liquiritigenin	0.19 ± 0.07	33.17 ± 11.99	0.12 ± 0.06	6.43 ± 2.42	286.43 ± 144.06	328.43 ± 198.16	8.85 ± 1.09	11.49 ± 3.02
Saikosaponin c	0.42 ± 0.09	0.96 ± 0.33	0.22 ± 0.11	4.23 ± 2.83	5.61 ± 1.92	5.93 ± 2.19	6.49 ± 1.17	7.67 ± 2.70
Glycyrrhizic acid	0.33 ± 0.11	18.65 ± 6.61	0.12 ± 0.05	7.63 ± 5.35	138.46 ± 56.98	156.17 ± 59.56	10.06 ± 0.58	13.61 ± 5.72
Saikosaponin a	0.42 ± 0.09	6.05 ± 1.30	0.12 ± 0.03	6.46 ± 2.57	16.26 ± 5.15	18.00 ± 6.88	6.61 ± 1.12	8.88 ± 3.49

C_max_: the maximum plasma concentration. t_max_: time to reach the maximum concentration. t_1/2_: half-life. AUC: area under the concentration-time curve. Kel: Elimination rate constant.

**Table 6:** Precursor/product ion pairs and parameters for MRM of compounds used in this study.

Analytes	CAS No.	Retention Time (min)	Precursor Ion Species	MRM Transitions Precursor Ion → Product Ion (*m*/*z*)	Cone Voltage (V)	Collision Energy (eV)
Albiflorin	39011-90-0	2.94	481.50 [M + H]^+^	481.50 → 104.90	10.00	20.00
Paeoniflorin	23180-57-6	3.23	497.90 [M + NH_3_]^+^	497.90 → 178.90	25.00	30.00
Ferulic acid	1135-24-6	3.57	195.00 [M + H]^+^	195.00 → 145.00	25.00	20.00
Senkyunolide I	94596-28-8	4.97	225.10 [M + H]^+^	225.10 → 207.00	10.00	20.00
Quercetin	117-39-5	5.10	303.20 [M + H]^+^	303.20 → 153.10	30.00	35.00
Isoliquiritigenin	961-29-5	7.23	257.00 [M + H]^+^	257.00 → 136.80	25.00	25.00
Atractylenolide III	73030-71-4	9.22	249.00 [M + H]^+^	249.00 → 231.00	15.00	10.00
Ligustilide	81944-09-4	10.22	191.20 [M + H]^+^	191.20 → 90.93	35.00	20.00
Atractylenolide II	73069-14-4	10.82	233.30 [M + H]^+^	233.30 → 91.00	25.00	30.00
Liquiritin	551-15-5	3.76	417.00 [M − H]^−^	417.00 → 255.00	35.00	20.00
Liquiritigenin	578-86-9	5.30	255.00 [M − H]^−^	255.00 → 135.00	25.00	30.00
Saikosaponin c	20736-08-7	7.55	971.60 [M + COOH]^−^	971.60 → 925.50	35.00	25.00
Glycyrrhizic acid	1405-86-3	8.14	821.00 [M − H]^−^	821.00 → 351.00	40.00	40.00
Saikosaponin a	20736-09-8	8.76	825.40 [M + COOH]^−^	825.40 → 779.60	35.00	45.00
Naringenin	480-41-1	4.43	273.10 [M + H]^+^	273.10 → 153.10	30.00	25.00
Naringenin	480-41-1	4.46	271.10 [M − H]^−^	271.10 → 151.10	30.00	25.00

MRM: multiple reaction monitoring.
